# Perspectives on Structural, Physiological, Cellular, and Molecular Responses to Desiccation in Resurrection Plants

**DOI:** 10.1155/2018/9464592

**Published:** 2018-06-25

**Authors:** Yathisha Neeragunda Shivaraj, Plancot Barbara, Bruno Gugi, Maïté Vicré-Gibouin, Azeddine Driouich, Sharatchandra Ramasandra Govind, Akash Devaraja, Yogendra Kambalagere

**Affiliations:** ^1^Centre for Bioinformation, Department of Studies and Research in Environmental Science, Tumkur University, Tumakuru 57210, India; ^2^Laboratoire de Glycobiologie et Matrice Extracellulaire Végétale, Normandie Univ, UniRouen, 76000 Rouen, France; ^3^Fédération de Recherche “Normandie-Végétal”-FED 4277, 76000 Rouen, France; ^4^Department of Studies and Research in Environmental Science, Kuvempu University, Shankaraghatta, Shimoga 577451, India

## Abstract

Resurrection plants possess a unique ability to counteract desiccation stress. Desiccation tolerance (DT) is a very complex multigenic and multifactorial process comprising a combination of physiological, morphological, cellular, genomic, transcriptomic, proteomic, and metabolic processes. Modification in the sugar composition of the hemicellulosic fraction of the cell wall is detected during dehydration. An important change is a decrease of glucose in the hemicellulosic fraction during dehydration that can reflect a modification of the xyloglucan structure. The expansins might also be involved in cell wall flexibility during drying and disrupt hydrogen bonds between polymers during rehydration of the cell wall. Cleavages by xyloglucan-modifying enzymes release the tightly bound xyloglucan-cellulose network, thus increasing cell wall flexibility required for cell wall folding upon desiccation. Changes in hydroxyproline-rich glycoproteins (HRGPs) such as arabinogalactan proteins (AGPs) are also observed during desiccation and rehydration processes. It has also been observed that significant alterations in the process of photosynthesis and photosystem (PS) II activity along with changes in the antioxidant enzyme system also increased the cell wall and membrane fluidity resulting in DT. Similarly, recent data show a major role of ABA, LEA proteins, and small regulatory RNA in regulating DT responses. Current progress in “-*omic*” technologies has enabled quantitative monitoring of the plethora of biological molecules in a high throughput routine, making it possible to compare their levels between desiccation-sensitive and DT species. In this review, we present a comprehensive overview of structural, physiological, cellular, molecular, and global responses involved in desiccation tolerance.

## 1. Introduction to Desiccation Tolerance

Plants are sedentary and are most vulnerable to extreme weather conditions especially desiccation (loss of cellular water content equivalent to air dryness) and their associated stress. Among various abiotic stresses encountered by plants, desiccation or dehydration stress due to water deficiency that causes metabolic disruption and mechanical damage to membranes is the most prominent [1, 2]. Desiccation tolerant plants can withstand drying equal to or below that of the absolute water content which is equal to that of complete dryness. Further, these resume normal biological function upon rehydration [3]. Some members of microbial, fungal, and plant kingdoms have evolved DT traits [3, 4]. However, this phenomenon is not common. Some of the land plants possess tolerance to desiccation in vegetative tissues [5] and are called resurrection plants. In the plant kingdom, DT is noted in most of the taxonomic groups ranging from pteridophytes to dicotyledons but absent in gymnosperms [3, 6]. The mechanism of survival under the extreme environmental fluctuations is highly complex, and not all plants have the ability to withstand desiccation. Most important creation that DT plants have to satisfy is to limit the damage to a repairable level. Also, physiological integrity should remain intact under dehydration, and repair mechanisms must exist upon rehydration [7]. Resurrection plants are deemed to be an excellent model to study the mechanisms associated with DT. DT species have been discovered in places with seasonally limited water availability and unreliable rainfalls; they also inhabit soils with minimal water retention as outcrops of rock at low to moderate elevations in tropical and subtropical zones. They have been collected in all the continents [8]. In total, only about 1,300 species of vascular plants are available, of which 135 are flowering plants (termed “resurrection plants”) and display desiccation tolerance in their vegetative tissues [6, 8] The majority of DT plants grow in the tropics and subtropics of East-West Africa and Southern Africa (including Madagascar), Brazil, Australia, North America, and the Western and Eastern Ghats in India [9].

The earliest terrestrial plants were desiccation tolerant at all stages of their life cycle [10]. However, in more complex vascular plants, DT has been retained in only reproductive tissues, but not in vegetative tissues [11]. DT plants today are able to produce vegetative structures like spores, seeds, and pollen, which can keep them viable in the desiccated state for decades, or centuries, for example, the ancient *Nelumbo nucifera* seed from China [12]. The mechanisms of DT in lower-order resurrection plants like algae, lichens, and bryophytes are not similar to those of angiosperms [13, 14].

The genetic mechanisms required for DT are not only exclusive to resurrection plants but also present in desiccation-sensitive (DS) plants [15]. However, resurrection plants express these genes not only in seed tissues but also in vegetative tissues which help these plants to survive desiccation [16]. For instance, genes encoding LEA (late embryogenesis abundant) proteins that are generally present in the seeds of DS plants at the time of embryo maturation [17] have been isolated from the desiccated vegetative tissues of resurrection plants like *C. plantagineum* [2] and *S. stapfianus* [18]. Current phylogenetic data suggest that vascular plants gained the ability to survive desiccation of their vegetative tissues through a mechanism that was first present in spores, and such an evolution has been identified in at least ten independent events within the angiosperms [10]. In addition to angiosperms, DT exists in pteridophytes, mainly *Selaginella* (*Selaginella*, Selaginellaceae, class Lycopsida, and order Selaginellales) and its species. *Selaginella* is an ancient group of lycophytes, a monophyletic equivalent of other vascular plants such as monilophytes (ferns *Psilotum* and *Equisetum*) and seed plants (gymnosperms and angiosperms) [19]. The single genus of *Selaginella* encompasses about 700 species, characterized by strongly flattened, frond-like branching and dimorphic leaves (microphylls) [20]. Some of the DT species of *Selaginella* are *lepidophylla* [21], *tamariscina* [22], and *bryopteris* [23].

**Figure 1:**
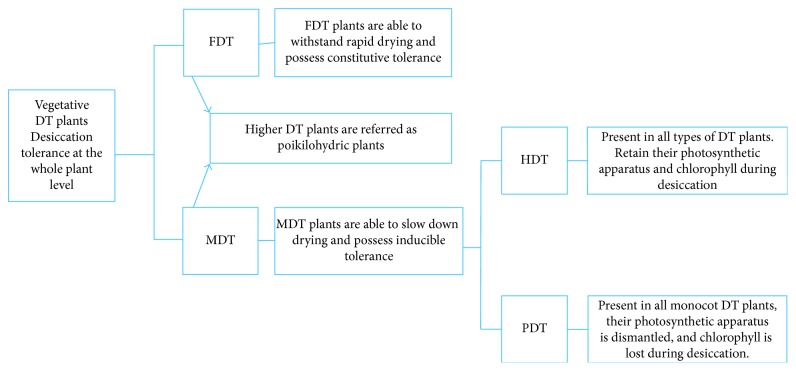
Classification of DT plants according to their stress adaptation strategies.

DT plants are classified based on the stress adaptation strategies which are shown in Table 1. They are divided into poikilochlorophyllous desiccation-tolerant (PDT) and homoiochlorophyllous desiccation-tolerant (HDT) plant (Figure 1) [39] types based on the status of the photosynthetic apparatus when dehydrated. During desiccation, HDT species retain their chlorophylls and photosynthetic apparatus in the readily recoverable state; for example, *Craterostigma* spp. retains the thylakoid and chlorophyll membranes intact through desiccation, although changes in photosynthetic pigment distribution were ascertained [40]. The chloroplasts of these plants have a distinct morphology including the round structure with an elaborate internal membrane organization. During the drying phase in homoiochlorophyllous vascular plants, the photochemical activity is much higher than CO_2_ absorption [41, 42]. Although the carbon fixation is suppressed through drying, the photoexcitation of chlorophyll in charge of the generation of ROS endures [43]. In *C. wilmsii* and *M. flabellifolius*, the photosynthesis is turned off during drying, via chlorophyll shading by leaf folding and anthocyanin accumulation [25, 44]. In HDT plants, rehydration can occur in a leaf disc that is detached from the plant or in a single leaf.

In case of PDT species, chlorophyll loss takes place as a result of desiccation, which is then recovered following rehydration [45]. For example, in *Xerophyta,* both chlorophyll and photosystem complexes are broken down and the thylakoid membranes are dismantled during the desiccation [44]. Accumulation of toxic ROS is lowered due to the degradation of chlorophyll, and it is an advantage in these plants. Since the chloroplasts lose chlorophylls, the entire thylakoid system, and most carotenoids during dehydration, the entire photosynthetic apparatus has to be reconstructed after rehydration [45, 46]. Loss of pigment and other thylakoid pigments destruction are highly organized in responses to desiccation which occurs through a well-defined metabolic pathway [45]. Thus, homoiochlorophyllous plant species resume photosynthesis faster than poikilochlorophyllous species which need to synthesize all components *de novo*. When poikilochlorophyllous leaves are detached from the plant, they cannot resurrect in contrast to the leaves of homoiochlorophyllous plants [47].

Taxonomically, PDT are reported from monocots [48]. Poikilochlorophylly is currently well known in eight genera of four families (Liliaceae/Anthericaceae, Cyperaceae, Poaceae, and Velloziaceae). Most of these plants occupy soil less rocky outcrops known as inselbergs in intensely seasonal subtropical and tropical climatic conditions [8]. Well-studied examples include the Australian *Borya nitid*, an African *X. viscosa*, *X. scabrida*, and *X. humilis* [49]. DT plants can also be subdivided according to the differences in the molecular mechanism of DT. Fully desiccation-tolerant (FDT) plants are capable of withstanding rapid drying and possess constitutive tolerance, while modified desiccation-tolerant (MDT) species have the capacity to survive slow drying and possess inducible tolerance [4, 50].

Some DT monocots developed the strategy of poikilochlorophylly to remain alive and participate in minimal habitats where availability of light is variable [11]. During drying, photosynthetic mechanisms in DT bryophytes are protected, which recovers rapidly resulting in rehydration [10]. The DT grass *S. stapfianus* is moderately poikilochlorophyllous and retains most of its chlorophyll content during desiccation [117]. Subsequently, a sequence of events takes place: they are dehydration to aridness and rehydration to complete turgor, and *S. stapfianus* recaptured all of its photosynthetic ability within 24 hours like other plants. The PSII levels in *S. stapfianus* demonstrated a definite decrease during the dehydration stage. This might be due to the fact that increasing water stress reduces the rate of photosynthesis. In order to retain membrane integrity and protoplast survival by a protective mechanism, toxic oxygen production is carried out by weakened PSII [51]. But only proteins inside the thylakoid membranes of resurrection plants stay constant during rehydration and desiccation; however, DS plants are entirely damaged after a short-term desiccation process [52].

## 2. Mechanism of Desiccation Tolerance in Resurrection Plants

DT is a very complex multigenic and multifactorial process comprising a combination of structural changes and macromolecular processes [53]. It is not an easy task for organisms to stay alive after losing their 90% of cellular water upon dehydration and to regrow when rehydrated. Cellular damage probability increases during desiccation in resurrection plants, so these plants survive by induction of mechanisms for protection and maintenance of cell integrity [54].

### 2.1. Leaf Structural Alterations

The scarcity of water leads to changes in morphological traits of resurrection plants. Morphological changes occur in vegetative tissues upon desiccation, and the most important is leaf folding [55]. Folding of leaves during drying occurs in both DT and DS plants. DT dicot leaves of *C. wilmsii* are completely expanded under well-watered conditions but gradually curl inward during drying and become firmly folded which facilitates the abaxial surfaces of mature leaves exposed to the sun [42]. These changes in the leaf shrink the transpiring surface and minimize oxidative stress damage by ultraviolet radiation [37, 55, 56]. The leaf blades of the DT monocot *X. humilis* bend into half along the midrib upon dehydration, leaving only the abaxial leaf surface exposed to the sunlight [56]. In the DT grass *S. stapfianus*, the adaxial side of the leaf rich in the epicuticular wax is exposed to the sun to limit heating of leaf tissues and irradiation [57]. During dehydration, two main events are held simultaneously: cuticular wax covering and closure of stomata, which helps to reduce the rate of water loss from the thylakoid membranes [57]. Closing stomata plants is most likely to minimize the loss of water through transpiration, and hence, the water flux through the plant is limited by reducing leaf growth, which creates a smaller transpiring leaf area as in *C. wilmsii*, *S. stapfianus*, and *X. humilis* [27, 58].

### 2.2. Cell Wall Involvement during Desiccation

The cell wall is a highly dynamic compartment that evolves during cell growth and cell differentiation and in response to biotic and abiotic stresses. The cell wall provides a protective barrier and consists mainly of cellulose microfibrils, hemicelluloses, pectins, and “structural” glycoproteins such as extensin and arabinogalactan proteins [59–61]. Plant cell walls are divided into a primary cell wall and a secondary cell wall. The primary cell wall is present in almost all cells of the plants, whereas the secondary wall is visible only in differentiated tissues. Moreover, two types of cell walls can be differentiated. Type I primary walls found in the eudicotyledons, noncommon in monocotyledons and gymnosperms [62], are composed of a network of cellulose microfibrils, mostly cross-linked with xyloglucans (XyG) and embedded in a matrix of pectic polysaccharides. Type II primary cell walls, characteristic of monocotyledons (grasses and rushes), are composed of glucuronoarabinoxylans (GAXs) and mixed-linkage (1→3),(1→4)-*β*-D-glucan (*β*-glucan) polymers that link cellulose microfibrils [63, 64]. Pectic polysaccharides and XyG are generally poorly represented in type II primary cell walls. Furthermore, ferulic and *p*-coumaric acid arabinosyl esters can cross-link GAX in type II primary walls.

Mechanical stress is one of the more challenging stresses that resurrection plants have to overcome in order to survive desiccation [65]. As water is lost from the cell, plasmolysis occurs resulting in plasma membrane tearing from the more rigid cell wall and cell death. Resurrection plants have developed strategies to minimize the impact of mechanical stress during desiccation and to avoid irreversible damages [66]. Indeed, during the dehydration of the resurrection plant *C. wilmsii*, a decrease of about 78% of the cellular volume occurs in foliar tissues [44]. This extensive reduction of mesophyll cells is due to a strong folding of cell walls. In *S. lepidophylla*, an important event of folding of the cell walls and plasmalemma with continuous apposition to the cell wall is visualized during desiccation [67]. In *M. flabellifolius*, the folding of the cell wall is less distinct, and it is not observed in all cell types [44]. The folding of the cell wall is considered as a strategy developed by cells of DT plants to maintain the contacts between the plasma membrane and the cell wall during dehydration and to avoid the tearing between these structures and hence cell lysis and death. Cell wall modifications do occur in DT plants in response to dehydration. These are listed in Table 2.

#### 2.2.1. Role of Cell Wall Cellulose in Desiccation Tolerance

One of the major constituents of both types of cell walls is cellulose, which exists as microfibrils composed of *β*-1,4-linked glucan chains that are linked by hydrogen bonds [78]. Cellulose is synthesized at the plasma membrane by a large multisubunit complex termed the “cellulose synthase” (CESA). The CESA is composed of at least three types of glycosyltransferases arranged into a hexameric rosette [78]. Although the protective function of cellulose during water stress has been well studied, there is no information available so far regarding the function of cellulose during desiccation. Some experiments reported a decrease of cellulose synthesis in response to water stress [79]. For example, in a switchgrass (*Panicum virgatum* L.), the transcript levels of *CesA1*, *CesA6*, and *CesA12* encoding a cellulose synthase were suppressed in response to drought stress. However, this effect is reversed upon rehydration [80]. It was proposed that cellulose synthesis is redirected in adapted cells to produce a hemicellulosic compound [81]. In contrast, in other studies carried out on cotton fibers, the abundance of the SuSy, UDP-Glc, and UGPase was enhanced under drought stress. This phenomenon indicates that cotton fibers are able to produce relatively more UDP-Glc for cellulose synthesis under drought stress.

#### 2.2.2. Role of Cell Wall Hemicellulose in Desiccation Tolerance

Hemicelluloses are important polysaccharides in plant cell walls consisting of *β*-(1→4)-linked backbones. Hemicelluloses include XyG, xylans, mannans, glucomannans, and *β*-(1→3, 1→4)-glucans. XyG are the major hemicellulosic polymers of dicot plants that strengthen the cell wall by forming a network with cellulose fibrils [87]. Existing models suggest the binding of each XyG polymer to at least two cellulose fibers [82]. This interaction can be modulated by two groups of enzymes: expansins and XyG endotransglucosylase/hydrolases (XTHs). In *C. wilmsii*, the structure of XyG, the major hemicellulosic compound, is affected by desiccation [59, 83]. Indeed, immunochemical studies revealed an increase of epitopes recognized by the XyG-directed monoclonal antibodies during dehydration. Furthermore, a modification in the sugar composition of the hemicellulosic fraction is detected during dehydration. An important decrease of the glucose in the hemicellulosic fraction during dehydration can reflect a modification of the XyG structure [83]. Expansins are another cell wall protein involved in remodelling of the cell wall. In the related species *C. plantagineum*, upregulation of gene expressions corresponding to expansin and xyloglucosyl transferase was closely correlated. The expansins might be involved in cell wall flexibility during drying and disrupt hydrogen bonds between polymers in the rehydration of the cell wall [24, 60]. Numerous studies were carried out on cell wall XTH and expansin. In the resurrection plant *A. rhodopensis* endemic to the Balkan area, upregulation of the transcript *Hrh*DR35 encoding an XTH was observed. This high level of the transcript was induced during the early stage of dehydration and persists in the desiccated state [31]. Another study revealed that overexpression of an *XTH* gene from hot pepper (*CaXTH3*) in transgenic *Arabidopsis* plants confirmed the role of XTH in drought tolerance. However, an abnormal leaf morphology resulting in a severely wrinkled leaf shape along with an irregular pattern of leaf cells was observed in response of the transgenic plants to dehydration. A similar study performed on transgenic tomato plants overexpressing the *XTH* (*CaXTH3*) showed that overexpression was able to confer tolerance under severe water-deficit conditions [84]. These results suggest that *CaXTH3* may be involved in cell wall remodelling, allowing the protection of the mesophyll cells from full dehydration. Another study suggests that the overexpression of *RhEXPA4* in *Arabidopsis* transgenic plants conferred a dehydration tolerance during the expansion of rose petals [85]. In similar experiments, expansin was overexpressed in tobacco, confirming its role in plant water retention ability and osmotic potential [86]. Together, these data suggest that cleavages by XyG-modifying enzymes release the tightly bound XyG-cellulose network, thus increasing cell wall flexibility required for cell wall folding upon desiccation.

Xylans are one of the major hemicelluloses in the secondary cell walls of dicots and all walls of grasses [87]. Xylans are a diverse group of polysaccharides with a backbone composed of *β*-(1→4)-linked xylose residues. These xylans usually contain many arabinose residues attached to the backbone and are known as arabinoxylans and GAXs [88]. The DT plants identified as the grass-like *Xerophyte* sp. and the grass *E. nindensis* are found to be enriched in arabinose and xylose, suggesting that arabinoxylans are major cell wall components in this species [61, 70, 89]. One can expect that arabinoxylans and xylans are involved in the regulation of mechanical properties of the cell wall during dehydration of *E. nindensis*. Ferulic acid can cross-link neighbouring arabinoxylan molecules or arabinoxylans to enhance cell wall stiffening [71]. Moreover, xylans are known to cross-link cellulose microfibrils, thus contributing to cell wall mechanical properties. The presence of arabinose substitution on the xylan backbone hinders hydrogen bonding between xylan chains and/or the cross-linking between xylans and cellulose microfibrils [90]. In *X. humilis*, significant differences are observed for arabinose substitutions between hydrated and desiccated leaf compositions and could indicate that dehydration may cause an increase in the wall arabinoxylan content and/or arabinosylation of wall xylans in this species [61].

#### 2.2.3. Role of Cell Wall Pectic Polysaccharides in Desiccation Tolerance

Pectins are the most complex family of cell wall polysaccharides. These components are enriched in galacturonic acid including homogalacturonan (HG), xylogalacturonan (XGA), rhamnogalacturonan I (RGI), and rhamnogalacturonan II (RGII) [91]. HG is the most abundant polysaccharide and represents 65% of the pectin, while RGI constitutes 20% to 35% [92]. XGA and RGII are minor components, each constituting less than 10% of pectins [92, 93]. HG is composed of free carboxyl groups that are able to cross-link with Ca2^+^ leading to the formation of a pectic gel [94]. HG can be methyl esterified through the action of PME (pectin methylesterase), which results in contiguous and random patterns of free carboxylic residues [95]. The PME enzyme activity is modulated specifically by inhibitor proteins such as the PME inhibitor (PMEI) [96].

Cell wall composition from the hydrated and dehydrated resurrection plants *C. wilmsii* and *C. plantagineum* shows a marked reduction of the demethylesterification of HG in the dry state [61]. A previous study showed that the overexpression of a pepper PMEI protein in *Arabidopsis* may be involved in drought stress tolerance [96]. Another experiment showed that the overexpression of stress-inducible *OsBURP16* in transgenic rice plants, which encodes the beta-subunit of polygalacturonase 1, induces a decrease of pectin content accompanied by sensitivity to drought compared to the wild-type [36]. *M. flabellifolius* exhibits an unusual arabinose-enriched primary cell wall. However, the monosaccharide composition of the cell wall remained unchanged upon desiccation [27, 61, 73]. It was hypothesized that the high content of arabinan was associated with RGI and/or arabinogalactan proteins. In the roots of cv. Capeiti, ‘‘drought-tolerant,” the number of side chains of RGI and/or RGII significantly increased in response to water stress. One of the possibilities is that these polymers would act as pectin plasticizers and could provide constitutive flexibility of cell walls, protecting the cell walls against water loss [73, 97].

### 2.3. Modification of Cell Wall Proteins during Desiccation Tolerance

#### 2.3.1. Hydroxyproline-Rich Glycoproteins (HRGPs)

Plant hydroxyproline-rich glycoproteins (HRGPs) are the major structural proteins in the cell wall [98, 99]. The HRGP family including arabinogalactan proteins (AGPs) and extensins (EXTs) consists of highly *O*-glycosylated proteoglycans [100]. AGPs are glycosylated with approximately 90% of carbohydrate moieties and only 10% of protein compounds of highly varying length. AGPs consist predominantly of arabinose and galactose residues, although other “minor” sugars including rhamnose, fucose, glucuronic acid, and xylose are also present [101]. AGPs are found inserted into the plasma in the external leaflet of the plasma membrane via a GPI anchor [101]. AGPs are known to be involved in many biological processes including cell development, cell death, cell-to-cell signalling, and cell defence [100]. Moore et al. proposed a model summarizing the importance of “plasticising” components of the cell walls in *M. flabellifolius* species during desiccation. AGPs are supposed to contribute to the flexibility of the cell walls during dehydration, and consequently, rehydration is facilitated in this plant [74]. The role of AGPs as pectic plasticizers is reviewed in [102], where they are shown to play a role in response to osmotic stress (i.e., *salt*). A study carried out on rice (*Oryza sativa* L.) showed that the expression of AGPs encoding genes was modulated in response to drought or salt stress. The expression of two AGPs genes, namely, *OsAGP3* and*OsAGP24*, was upregulated in response to drought stress, whereas the expression of three other genes (*OsELA3*, *OsAGP1*, and *OsAGP25*) was upregulated in response to both drought and salt stresses [103]. The authors hypothesized that deglycosylation of AGPs by glycosidases would result in the release of oligosaccharides, which in turn would increase the intracellular osmotic pressure to reduce the rate of dehydration [103].

EXTs contain mainly hydroxyproline (Hyp), but serine (Ser), lysine (Lys), tyrosine (Tyr), histidine (His), valine (Val), and alanine (Ala) are also constitutive of the protein. The repetitive pattern (Ser-(Hyp)_4_) and the sequence (Tyr-Lys-Tyr) are characteristic of EXTs [99, 104, 105]. EXTs consist of arabinoside chains limited to 4-5 arabinosyl residues on Hyp residues and single galactose residues attached to Ser residues. EXTs have been extensively studied and shown to fulfill functions related to abiotic stresses in plants [106]. However, there is no information available so far regarding the EXT function during desiccation. Studies carried out on two potato clones (*Solanum tuberosum*) of the Andean cultivar group with different drought-tolerant phenotypes showed that transcription of one EXT gene was induced in both cultivars [107]. The transcript EXT3 was also shown to be upregulated under mild drought in *Arabidopsis*. This upregulation was accompanied by loosening of the cell wall allowing for a reduced growth under lower turgor [108].

#### 2.3.2. Role of Glycine-Rich Proteins (GRPs) in Desiccation Tolerance

In plants, glycine-rich proteins (GRPs) are characterized by the presence of more than 60% glycine [109]. There are five classes of GRPs: three classes are based on the pattern of the glycine-rich repeats (class I, GGGX; class II, GGXXXGG; and class III, GXGX) and two other classes are based on the type of functionally conserved motifs (class IV, the oleosin glycine-rich proteins, and class V, the RNA-binding GRPs). Most of the GRPs known to date have been found in the cell walls of many higher plants and form another group of “structural proteins” of the cell wall [110].

GRPs are known to be modulated by abiotic factors. By analogy to HRGPs, those proteins have been proposed to act as a scaffold or agglutinating agents for deposition of cell wall constituents [111]. Analysis of transcripts expressed in desiccated leaves of *C. plantagineum* identified a gene putatively coding for an apoplastic glycine-rich protein (CpGRP1). CpGRP1 interacts with CpWAK1 which is downregulated in response to dehydration. The CpGRP1-CpWAK1 complex could be inducing morphological changes in the cell wall during dehydration in *C. plantagineum*. In fact, cell wall pectins and dehydration-induced pectin modifications are predicted to be involved in the activity of the CpGRP1-CpWAK1 complex [69].

### 2.4. Changes in the Antioxidant Systems during Desiccation Tolerance

During dehydration, resurrection plants produce high amounts of antioxidants [28]. The desiccation process can damage membrane lipids and proteins by producing a number of reactive oxygen species (ROS). By using an effective mechanism, *S. stapfianus* stimulates free radical scavenging enzymes, for example, ascorbate peroxidase, dehydroascorbate reductase, and glutathione reductase, to eliminate the ROS [112]. It has been shown that more injury occurs during rehydration than in desiccation because of increased oxidative stress at the time of the recovery phase [112]. Desiccation enhances the antioxidant activity in other resurrection plants also [42, 113]. In the DS plant *S. stapfianus*, when leaves are dried and detached, ascorbate peroxidase activity is decreased which leads to a reduction in antioxidant capacity [112].

The antioxidant enzymes superoxide dismutase (SOD), glutathione reductase (GR), catalase (CAT), and ascorbate peroxidase (APX) show a good response desiccation in DS and DT organisms. However, the response is significantly enhanced during desiccation in DT plants [16]. This phenomenon of higher induced antioxidant enzyme activity is a distinct DT mechanism [16]. Resurrection plants are able to sustain in the desiccated state for many years in relation to DS plants because increases in duration of desiccation will lead to more oxidative damage with gradual loss of the acyl chains of membrane polar lipids. Furthermore, the membranes contain unsaturated double bonds, very sensitive to free radical attack. Resurrection plants contain more number of double bonds in their polar lipids, which is a common characteristic of chloroplasts [113]. The antioxidant system comprising a number of enzymes fails during long duration due to programmed cell death, triggering aging and ultimately plant death as in DS plants [16]. It has been demonstrated that there is a negative association between the longevity of DT tissues and the number of double bonds in the polar lipids of the membranes, which ultimately determines how long resurrection plants are able to survive desiccation.

### 2.5. Cellular Membranes Lipid Composition Changes during Desiccation Tolerance

In the DT mosses *S. lepidophylla* and *T. ruralis*, dehydration results in cell shrinkage leading to highly convoluted membranes and walls [38]. During desiccation, the plasma membrane contains numerous tightly associated lipid droplets similar to a normal lipid bilayer organization. Desiccation normally causes unsaturation, and the level of individual phospholipids within the total lipids decreases in DT vascular plants [114]. However, unusual trends were noticed in the DT plant *B. hygroscopica* where an increase in unsaturation of all classes of fatty acids during desiccation was observed [115]. It is well known that a high degree of polyunsaturation in phospholipids is responsible for greater membrane fluidity. An increase in membrane fluidity is an important phenomenon for the survival of desiccation. The phospholipid content increases in leaves which are dried and attached to the parent. However, it decreased in leaves that are dried and detached in *S. stapfianus* during desiccation [116]. The dried leaves attached to the parent improve desiccation tolerance because of an increase in polyunsaturated fatty acids within the plasma membrane. However, in detached and dried leaves, this is not true, and hence, desiccation sensitivity might increase [17]. In *S. stapfianus*, leaves desiccated on the plant regained almost all of the lipid content, whereas detached dried leaves suffered complete lipid degradation with the loss of polyunsaturated fatty acids when rehydrated [116].

### 2.6. Signalling Mechanisms Involved in Desiccation Tolerance

During desiccation stress, an increase in the concentration of secondary messengers helps to control the intracellular Ca^2+^. This results in protein phosphorylation and transcription of stress-controlled genes. Plants under desiccation stress show a marked increase in ABA accumulation as one of the early responses. ABA plays a key role in the initiation of DT resulting in expression of proteins [117, 118]. Several dehydration-regulating genes are linked to ABA in DT plants [72, 119–121]. Regulatory pathways and gene signalling information in resurrection plants are relatively unknown in comparison with *Arabidopsis*. Phospholipid-based signalling in *C. plantagineum* is the main primary signalling pathway responsible for downstream mitigation of desiccation [122]. Dehydration induces the activity of two cDNA clones encoding phospholipase D in *C. plantagineum* but not that of ABA. By generating CpPLD-1 transcripts, secondary messenger molecules are engaged in primary reactions to dehydration. Furthermore, coexpression of CpPLD-2 results in an enhanced metabolism of phospholipids [122]. Dehydration-induced transcription factors regulated by phosphor lipases in *C. plantagineum* include the myeloblastosis family [123], basic leucine zipper family [124], homeodomain-leucine zipper family [125, 126], and a novel zinc finger factor [127].

### 2.7. Role of LEA Proteins in Desiccation Tolerance

LEA proteins accumulate to very high levels during the late stage of embryogenesis in seeds when they dry [128]. They also accumulate during dehydration in reproductive and vegetative tissues of plants. Therefore, LEA proteins are adaptive in nature and help to counter cold, drought, dehydration, desiccation, and salt in vegetative tissues [129]. They are also known to respond to ABA, and the plants with a high degree of LEA expression survive desiccation better [130]. Important roles of LEA proteins include DNA repair or unwinding, counteracting the physical stresses imposed from desiccation by stabilizing the filaments of cytoskeletons and chaperone activity to guard protein activity and its conformation [131, 132]. It has also been noticed that these proteins can act collaboratively with sugars, like trehalose, for preventing the aggregation of protein during desiccation [133]. Most of the LEA proteins belong to a more well-known group of proteins called “hydrophilins” characterized by 6% glycine and a high hydrophilicity index making them soluble at high (80°C) temperatures. LEA proteins are ubiquitous in the plant kingdom which are present not only in gymnosperms and angiosperms but also in seedless vascular plants and even in algae, bryophytes, and pteridophytes [54]. Also, these proteins are present in yeasts, bacteria [134, 135], nematodes [136, 137], and fungi [138, 139]. It has also been shown that ABA regulates the LEA protein expression along with dehydration-induced genes [72]. In *C. plantagineum*, a minimum of two LEA proteins (CDeT 11–24 and CDeT 6–19) are phosphorylated *in vivo* during desiccation [140]. Similar to LEA proteins, some other molecules like polyphenols (gallolylquinic acids) and small heat shock proteins are also responsive to desiccation [141]. These molecules safeguard membranes against desiccation, which shows that alternate novel elements with LEA like functions are active in resurrection plants. Reviews of Battaglia [142, 143] describe in detail the features and functions of the LEA proteins with respect to DT. LEA proteins perform a function in stabilizing membranes or in the transport of lipids for the reformation of damaged membranes in rehydrating *T. ruralis* gametophytes [10] or the transport of lipids for reconstitution of broken membranes [10]. Although a number of LEA genes have been isolated, a useful role in desiccation tolerance has been established for very few genes. The expression of the barley LEA sequence, HVA1, increased drought tolerance in transgenic wheat plants. Similarly, in rice, overexpression of HVA1 enhances tolerance to both drought and salt stress [144].

### 2.8. Role of Small Regulatory RNAs in Desiccation Tolerance

A significant role of small regulatory RNAs in monitoring the plant responses to desiccation stress is widely accepted in the recent scientific literature [119, 145, 146]. It has already been shown that the application of exogenous ABA in *C. plantagineum* callus was able to induce DT. Induction of DT was mediated by CDT-1 constitutive expression and other ABA-inducible genes. Other reports also suggest that constitutive expression of ABA-responsive transcripts in *C. plantagineum* is ABA independent [146]. Some other functionally related genes and CDT-1 members have abilities of a short interspersed element retrotransposon. Hence, it has been hypothesized to act as regulatory noncoding RNA molecules which are distinctive in *C. plantagineum* [147].

## 3. Functional *-omic* Studies on Desiccation Tolerance

The term “desiccomics” coined by [148] describes the combined -*omic* approaches to address and understand the global level changes associated with the dry state of the resurrection plants. Present-day progress in “-*omic*” technologies has enabled us in quantitative monitoring of the plethora of biological molecules in a high throughput routine, enabling comparison between desiccation-sensitive and desiccation-tolerant species. We summarize transcriptomic, proteomic, metabolomic, and genomic responses in various desiccation-tolerant plants below.

### 3.1. Transcriptome Analysis in Desiccation-Tolerant Plants

Thousands of ESTs (expressed sequence tags) can be analyzed through transcriptomic approaches. Expression profiling of transcriptomics (mRNA) can capture spatial and temporal gene expression while also quantifiying RNAs under different conditions. Either gene microarray techniques or quantitative reverse transcriptase polymerase chain reaction- (qRT-PCR-) based quantitative analysis of gene expression can be conducted. Current advances in assembly algorithms and sequencing technologies have enabled the reconstruction of the whole transcriptome via deep RNA sequencing (RNA-seq). Using these technologies, resurrection plants without any reference genome can also be analyzed.

In the studies mentioned above in Table 3, cDNA libraries for EST sequencing were generated in either single or two physiological situations (rehydrated and dehydrated fronds/roots/leaves or gametophytes). These studies reflect global transcript changes. Such integrated transcriptome analysis studies have so far been reported for *Haberlea rhodopensis* and *C. plantagineum* [24, 161].

The rehydrated moss *T. ruralis* was subjected to EST sequencing of a cDNA library which resulted in the identification of around 10,368 ESTs with 5,563 genes [152]. Transcriptomic studies of *H. rhodopensis* and *C. plantagineum* in different physiological stages (desiccated, control, rehydrated partially, and dehydrated) showed transcripts with the highest match to genes of *Vitis vinifera*, *Populus trichocarpa*, and *Ricinus communis*. In *C. plantagineum*, transcripts of 182 MB sequences were assembled into 29,000 contigs, which further produced 15,000 more unique individualities. Similar studies showed that 96,353 expressed transcript contigs of *H. rhodopensis* were characterized [24, 161]. Important knowledge generated from these studies shows that one-third of the contigs from *C. plantagineum* and around 40% contigs from *T. ruralis* and *H. rhodopensis* could not be mapped to UniProt identities. Thus, they are unknown transcripts which are possible sources for future gene detection. Depending on the expression patterns observed for *H. rhodopensis* and *C. plantagineum*, transcripts can be divided into two main groups. The first group contains transcripts abundant in control and rehydrated plant tissues, and the second group comprises abundant transcripts in dehydrated and desiccated plant tissues [24, 161].

### 3.2. Proteomics Studies in Desiccation-Tolerant Plants

Proteins play a major role in plant adjustment to desiccation stress as they are involved directly in the plant metabolism and cell structure. Proteins induced by desiccation consist of regulatory proteins (e.g., signalling proteins, protein kinases, transcription factors, and protein phosphatases). Also, effector proteins that are directly involved in acquisition of desiccation tolerance are LEA proteins, channel proteins, mRNA-binding proteins, components of protein biosynthesis and degradation, water osmolyte synthesis enzymes, detoxification enzymes, and cytoskeletal proteins. Owing to the low abundance of signalling proteins and transcription factors mentioned above, their protein complexes are not easily identified in various states of dehydration and rehydration by classical proteomics. Furthermore, to carry out a specific function, these proteins function as components of larger complexes, and these complexes may be regulated from the stage of its formation. Thus, characterizing these protein complexes will enable vital understanding of these proteins in different stages of desiccation/rehydration.

Reports of proteome analysis in resurrection plants are limited to a very few species. A direct association between protein and transcript richness has been recorded for several gene products with protective functions for dehydration [22, 29, 32, 162]. Quantitative proteome data correlate with transcript data which further confirms that the proteins associated with the carbohydrate metabolism and photosynthesis are abundant in the hydrated tissues of DT plants. Furthermore, termination of and reactivation of photosynthetic activity are major responses observed during desiccation and rehydration, respectively [22, 24, 32, 162].

The decline in the photosynthesis rate is proportional to reduction in abundance of chloroplast photosynthetic proteins, for example, psbO, psbP, the subunit of the F-ATPase, the PSII stability factor HCF136, and the transketolase [32]. In desiccation-tolerant resurrection plants, during desiccation, LEA proteins accumulate abundantly [117, 163–166]. In *C. plantagineum*, use of 2D-SDS-PAGES and phosphoprotein-specific stain shows that a minimum of two proteins were phosphorylated. Phosphorylation may possibly increase the hydrophilic residues which are necessary for interaction with other macromolecules. Phoshorylation is important for proper subcellular localization as was revealed for LEA proteins in the maize embryo. However, the role of phosphorylation in the LEA proteins CDeT11-24 and CDeT6-19 is yet to be discovered [167]. In resurrection plants, proteome analysis has shown the expression of unknown proteins. In case of *S. tamariscinia*, functions of 103 unique proteins from 138 protein spots responsive to dehydration could not be ascertained. During dehydration, proteins downregulated in *S. tamariscina* comprised those involved in the energy and carbohydrate metabolism, photosynthesis, stress signalling, membrane transport, defence proteins, cell division, and cell structure. However, protein abundance increased for antioxidant enzymes [22].

From the leaves of *B. hygrometrica*, 200 unique proteins were analyzed. Among these proteins, 35% (78) increased in response to desiccation stress, 60% showed decreased levels or remained unchanged, and 50% were induced under rehydration conditions. Many of the proteins associated with the antioxidant and energy metabolism were constitutively expressed which shows the occurrence of constitutive protective mechanisms. Proteins induced due to dehydration in *B. hygrometrica* are related to GSH, polyphenol metabolism, energy, and metabolism indicating that GSH serves as a key antioxidant. Furthermore, analysis of proteins indicates the photosynthetic degradation-related proteins like a 20 kDa fragment of the RuBisCO large subunit (RbcL) and an oxygen-evolving complex (a 23 kDa polypeptide of the photosystem II). A 20 kDa·RbcL was identified in dehydrated leaf proteins. Protein fragments appearing in *B. hygrometrica* are assumed to be the consequence of stress-related proteolysis rather than chloroplast-localized protease activity which is ROS-induced [15]. During dehydration, ATP-dependent transport of solutes mediated by ABC transporters was also induced in *B. hygrometrica* [29]. The putative induction of ATPase subunits identical to a vacuolar H^+^-ATPase A subunit on dehydration might help in rehydration preparation. Desiccated leaves of *X. viscosa* and *Sporobolus stapfianus* showed the same profile of protein as that of *B. hygrometrica* [168, 169]. Upregulation of enzymes is associated with sugar metabolism like ADP-glucose pyrophosphorylase, sucrose synthase, and GDP-mannose 3,5-epimerase, confirming the importance of sugar metabolism during desiccation stress. Protein expression patterns observed in different resurrection plants show that several proteins are rapidly and massively induced upon dehydration. These proteins continue to exist throughout the period of desiccation and carry out diverse functions like scavenging ROS, protecting proteins, sucrose accumulation, restoration of cell wall proteins and proteins also with unknown functions. Even though the *tkt3* transcript levels are expressed constitutively in vegetative tissues *of C. plantagineum*, in hydrated tissues, the corresponding protein levels are too high, suggesting a slower protein turnover or a high translation rate during hydration. Similarly, in *C. plantagineum*, *tkt7* mRNA abundance during late phases of rehydration does not match the protein abundance [168]. This phenomenon is also true for regulatory genes like transcription factors, which are difficult to investigate because of low abundance.

### 3.3. Metabolomic Analysis in Desiccation-Tolerant Plants

Metabolomic studies in resurrection plants deal with the quantitative analysis of small molecules in different metabolic states. Different study approaches like mass spectrometry (MS), gas chromatography (GC), liquid chromatography (LC), capillary electrophoresis, and nuclear magnetic resonance (NMR) spectroscopy are used to analyze a large variety of chemical structures. *Arabidopsis* is the choice model for metabolic studies because of its simple metabolism and the low rate of emission of metabolites during dehydration. Metabolomic studies on DT have been reported for two closely related *Sporobolus* and *Selaginella* species which differ in their desiccation tolerance [170, 171]. They showed that metabolite levels vary during desiccation/rehydration which includes lipids, nucleotide derivatives, carbohydrates, amino acids, polyamines, antioxidants, and defence compounds.

A number of significant metabolites have been identified in metabolomic studies on resurrection plants (Table 4). The metabolic state of fully hydrated *S. lepidophylla* was found to be different from the dehydration state. The mapping of the identified metabolites (66.5%) into the biochemical pathways shows that the dominant metabolites were amino acids (19%) followed by cofactors, carbohydrates, nucleotides, lipids, peptides, and secondary metabolites. However, peptides, amino acids, and nucleotide metabolites were more significant during desiccation. In the hydrated state, carbohydrates such as 4C–6C-containing sugars, lipids, or lipid metabolites (with the exception of choline phosphate), sugar alcohols, and cofactors were also more significant [176]. Among the 251 identified metabolites of *S. lepidophylla*, 33.4% were unknown metabolites. Seven unknown metabolites were of greater abundance in dehydration conditions than in hydrated states signifying their role in DT. Studies have shown that *S. stapfianus* metabolically prefers dehydration because of increased concentrations of nitrogen and osmolyte metabolites along with metabolites related to energy in the hydrated state. During dehydration, the metabolism moved towards carbohydrate and nitrogen remobilization, antioxidant production, and ammonia detoxification [162]. This also seems to be the case in *C. plantagineum* where major metabolite differences were predominantly during dehydration.

### 3.4. Genomic Studies on Desiccation-Tolerant Plants

The availability of genome sequences of DT plants though not on the scale of drought-tolerant plants has enabled a system-level effort to understand the complexities of DT [180]. Genomics is extremely important in order to complement transcriptomic and proteomic studies and also to get better expression profiles in response to desiccation [181]. Extensive studies on the genetic network activated in DT plants will help the scientific community to design mutants in order to evaluate the role of single/multiple gene(s) [182]. So far, genome sequences of only three DT species *O. thomaeum*, *B. hygrometrica*, and *X. viscosa* are available [183–185]. The curated data available do not seem to suggest any typical genomic features that are only specific to DT. Existence of co-linearity between genome structures in DT and non-DT plants shows that no specific genome-level effects occur because of desiccation, and examples of such a co-linearity are known in DT *O. thomaeum* and other grasses [181]. However, some important genes for protective proteins are present in duplicates indicating a transcribed genome [181]. Chloroplast genomes of *T. ruralis* are different from those of another moss *Physcomitrella patens*, both of which have different levels of sensitivity towards desiccation [175]. In addition, the whole-genome sequencing of the desiccation-tolerant grass *O. thomaeum* can serve as a valuable resource for the plant comparative genomics community [185]. Whole-genome sequence data of *X. viscosa* revealed that transcripts induced were typically desiccation tolerant in nature. Among the salient features of the genome during dehydration was reduced, transcript abundance of genomic “clusters of desiccation-associated genes” (CoDAGs), which might be due to complete stop of the growth that leads to an increase in expression of desiccation tolerance [183]. The genome of *B. hygrometrica* is approximately ∼1,548 MB in size. Approximately 85.86% of the assembly is a nongapped sequence. Gene prediction tools show that 49,374 protein-coding genes and 40.68% have been validated by RNA-Seq; among them, 23,250 (47.09%) were found to be similar to database entries resulting in assignment of gene function [184].

## 4. Conclusion

**Figure 2:**
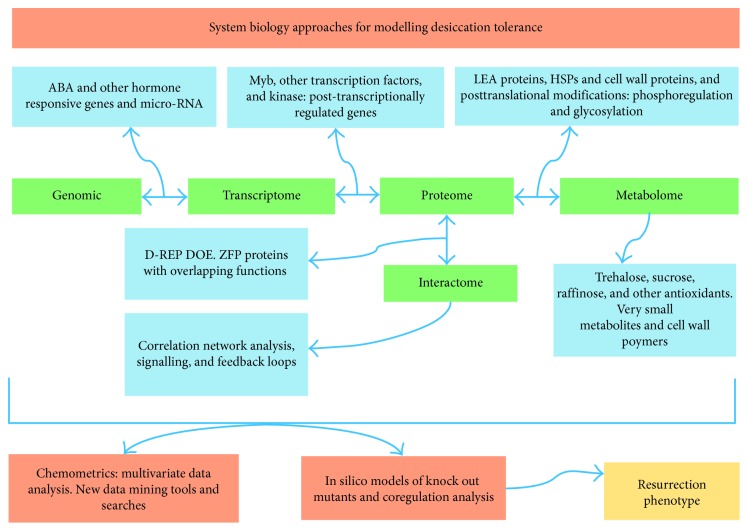
Systems biology framework for DT studies.

DT plants have extraordinary ability to regrow when rehydrated. This resilience is mainly because of their ability to alter the leaf structure and modify cell wall proteins and polymers during water loss and subsequent recovery. The recovery to the original morphology and physiological state is further aided by changes in photosynthesis, PSII activity, antioxidant systems, and lipid composition of cellular membranes. Also, ABA, LEA proteins, and small regulatory RNAs are responsible for regulating DT responses. Comprehensive and comparative information on changes in proteins/transcripts/metabolites in DT and DS species are now available mainly due to a recent surge in -*omic* technologies. However, the most challenging in terms of obtaining deep data coverage among these technologies is proteomics due to the low abundance of protein complexes. Recent improvements to various proteomic technologies have increased the sensitivity and robustness of protein identification during DT. In addition to these technological developments in peptide recovery and identification, further developments in bioinformatics and downstream validation technologies are required to make sense of complex data on DT. Therefore, in order to obtain more functional data on DT, a systems biology workflow with a specific focus on DT is the need of the hour. We therefore propose a new systems biology model (Figure 2) by integrating various functional -*omic* data sets with the aim of identifying new signalling intermediates and feedback loops responsible for overlapping/complex protein interactions resulting in desiccation tolerance. Implementing such a systems biology workflow will enable high confidence comparison of smaller proteomic data with those of the much larger microarray data sets. The strong and repetitive identification of low abundance proteins might just be possible with such a systems biology workflow which is a necessary prerequisite for analyzing global changes in DT plants comprehensively.

## Figures and Tables

**Table 1:** Classification of DT plants according to the types, family, class, and origin.

Name	Family	Class	Origin	HDT/PDT	References
*Craterostigma plantagineum*	Scrophulariaceae	Dicot	Southern Africa	H	[24]
*Craterostigma wilmsii*	Scrophulariaceae	Dicot	Southern Africa	H	[25]
*Lindernia brevidens*	Linderniaceae	Dicot	East Africa	H	[26]
*Myrothamnus flabellifolius*	Myrothamnaceae	Dicot	Southern Africa	H	[27, 28]
*Boea hygrometrica*	Gesneriaceae	Dicot	China	H	[29]
*Ramonda serbica*	Gesneriaceae	Dicot	Serbia	H	[30]
*Haberlea rhodopensis*	Gesneriaceae	Dicot	Balkan mountains	H	[31]
*Xerophyta viscosa*	Velloziaceae	Monocot	Southern Africa	P	[32]
*Xerophyta humilis*	Velloziaceae	Monocot	Southern Africa	P	[33]
*Sporobolus stapfianus*	Poaceae	Monocot	Southern Africa	—	[34]
*Eragrostis nindensis*	Poaceae	Monocot	Southern Africa	P	[35]
*Selaginella bryopteris*	Selaginellaceae	Lycophyte	India	H	[23]
*Selaginella tamariscina*	Selaginellaceae	Lycophyte	China	H	[36]
*Selaginella lepidophylla*	Selaginellaceae	Lycophyte	North and South America	H	[37]
*Tortula ruralis*	Pottiaceae	Bryophyte (moss)	North America	H	[38]

**Table 2:** Cell wall modifications of DT plants.

Name	Cell wall modification	References
*Boea hygrometrica*	(i) Extensive cell wall folding accompanied by protoplasmic shrinkage	[16, 22, 68]
	(ii) An increase of pectin and wax/suberin events occurred mainly during the rehydration phase	
	(iii) The contents of cell wall-associated lignin were reduced in desiccated leaves	
	(iv) Transcripts encoding cell metabolism were induced in rehydrated acclimated plants, indicating cell wall loosening during rehydration	

*Craterostigma plantagineum*	(i) A marked reduction of the demethylesterification of HG in the dry state	[24, 60, 61, 69]
	(ii) An upregulation of gene expressions corresponding to expansin and XyG synthesis	
	(iii) CpGRP1-CpWAK1 complex could be inducing morphological changes	
	(iv) A role for CpCRP1 in the leaf cell wall prior to dehydration stress and in mechanisms which are required for the successful recovery from desiccation	
	(v) The transcripts encoding proteins involved in ion transport such as membrane-associated carriers together with proteins involved in cell wall plasticity are abundant in fully hydrated conditions in *C. plantagineum*	

*Craterostigma wilmsii*	(i) Decrease about 78% of the cellular volume	[44, 59]
	(ii) A strong folding of the cell wall	
	(iii) A modification in the sugar composition of hemicellulosic fraction	
	(iv) An increase of epitopes recognized by the XyG-directed monoclonal antibodies	

*Eragrostis nindensis*	(i) Arabinoxylans and xylans are involved in the regulation of mechanical properties of cell walls	[61, 70, 71]
	(ii) Ferulic acid can cross-link neighbouring arabinoxylan molecules or arabinoxylans to enhance cell wall stiffening	

*Haberlea rhodopensis*	(i) Upregulated transcript *Hrh*DR35 encoding an XyG endotransglucosylase/hydrolase	[31, 72]
	(ii) Downregulation of many cell wall-related genes including XyG endotransglucosylases and pectate lyases	

*Lindernia brevidens*	(i) A strong folding of the cell wall	[26]

*Myrothamnus flabellifolius*	(i) Arabinose-enriched primary cell wall	[27, 61, 73, 74]
	(ii) AGP is a contributor in ensuring flexibility and to facilitate the rehydration	

*Ramonda serbica*	(i) Activities of nonspecific peroxidases play a role in cell wall remodelling	[75]

*Selaginella bryopteris*	(i) Phospholipase A1 gamma-like protein and glucan endo-1,3-alpha-glucosidase Agn1 have been reported to play a structural role in reinforcing the cell wall during stress	[76]

*Selaginella lepydophylla*	(i) A strong folding of the cell wall	[67]
	(ii) Plasmalemma with continuous apposition to the cell wall	

*Sporobolus stapfianus*	(i) A strong folding of the cell wall	[57, 77]
	(ii) Transcripts encoding enzymes involved in cell wall remodelling are increased in abundance during dehydration	
	(iii) A late accumulation of ferulate and caffeate, precursors of cell wall lignin and cross-linking compounds, could enhance cell wall extensibility	

*Xerophyta* spp.	(i) Highly arabinosylated xylans and arabinogalactan proteins	[61]

**Table 3:** Gene expression and EST sequencing studies on various resurrection plants.

Species name	Plant name	References
Moss species	*T. ruralis*	[149–152]
Clubmoss species	*S. lepidophylla*	[36, 123, 153]
	*S. tamariscina*	
Monocot species	*S. stapfianus*	[18, 154]
	*X. viscosa*	[155–157]
	*X. humilis*	[16, 33, 158]
	*X. villosa*	[159]
Dicot species	*C. plantagineum*	[160]

**Table 4:** 

Name of the metabolites	Role of metabolites during desiccation in plants	References
*Carbohydrates*: sucrose, raffinose, maltose, verbascose, stachyose, arbutin, glucosylglycerol, trehalose, and glucose	Replacing water on membranes and macromolecules by formation of anhydrous glass vitrification of the cytoplasm filling and stabilization of vacuoles and membrane proteins	[44, 52, 129, 172–175]

*Amino acids*: glutamate, glutamine, arginine, citrulline, aspartate, asparagine, *N*-6-trimethyllysine, and *trans*-4-hydroxyproline, and the intermediate metabolites 3-(3-hydroxyphenyl)propionate and the tripeptide ophthalmate (L-Y-glutamyl-L-*α*-aminobutyrylglycine), quinate, *γ*-glutamyl, tryptophan, and the derivatives acetyltryptophan or phenylalanine	Biosynthetic precursors for primary and secondary metabolites	[34, 161, 170, 176]
	These amino acids could function as compatible solutes or as mobile nitrogen reserves for the rehydrating tissues	
	Activation of the shikimate pathway which can result in the synthesis of antioxidants	

*Nucleotide metabolites*: allantoin, 1-methyladenosine, uridine 5′-monophosphate, and inosine	Plant stress protection by influencing ABA production, purine catabolism, and quenching ROS	[170, 176]

*Lipids*: phosphatidylinositol, phosphatidic acid, lysolipids, fatty acids, choline phosphate, and lipoxygenase	Maintenance of membrane integrity and maintenance of membrane fluidity to allow for recovery after dehydration	[122, 129, 176, 177]

*Polyamines*: spermidine and spermine	Membrane stabilization, enzyme activity modulation, plant growth and development, nitrogen assimilation, and respiratory metabolism. Protect ion of macromolecules	[178, 179]

*Antioxidants*: superoxide dismutase, catalase, ascorbate peroxidase, glutathione, etc.	Detoxify the ROS which arises during desiccation stress	[16, 156, 176]

## Abbreviations

ABA: Abscisic acid
AGPs: Arabinogalactan proteins
Ala: Alanine
APX: Ascorbate peroxidase
CESA: Cellulose synthase
DS: Desiccation sensitive
DT: Desiccation tolerance
EXTs: Extensins
FDT: Fully desiccation tolerant
GAX: Glucuronoarabinoxylans
GR: Glutathione reductase
GRPs: Glycine-rich proteins
HG: Homogalacturonan
His: Histidine
HRGPs: Hydroxyproline-rich glycoproteins
Hyp: Hydroxyproline
LEA: Late embryogenesis abundant
Lys: Lysine
MDT: Modified desiccation tolerant
PME: Pectin methylesterase
PMEI: PME inhibitor
PS: Photosystem
RGI: Rhamnogalacturonan
IRGII: Rhamnogalacturonan II
ROS: Reactive oxygen species
Ser: Serine
SOD: Superoxide dismutase
Tyr: Tyrosine
Val: Valine
XGA: Xylogalacturonan
XTH: XyG endotransglucosylase/hydrolase
XyG: Xyloglucans.
